# Green Synthesis of Hydrophobic Magnetite Nanoparticles Coated with Plant Extract and Their Application as Petroleum Oil Spill Collectors

**DOI:** 10.3390/nano8100855

**Published:** 2018-10-19

**Authors:** Mahmood M. S. Abdullah, Ayman M. Atta, Hamad A. Allohedan, Hamad Z. Alkhathlan, M. Khan, Abdelrahman O. Ezzat

**Affiliations:** 1 Department of Chemistry, College of Science, King Saud University, P.O. Box 2455, Riyadh 11451, Saudi Arabia; altaiar90@yahoo.com (M.M.S.A.); hlohedan@ksu.edu.sa (H.A.A.); khathlan@ksu.edu.sa (H.Z.A.); mkhan3@ksu.edu.sa (M.K.); ao_ezzat@yahoo.com (A.O.E.); 2 Department of Chemistry, Faculty of Applied Science, University of Taiz, P.O. Box 4007, Taiz, Yemen

**Keywords:** green synthesis, plant extract, *Anthemis pseudocotula*, magnetite nanoparticles, oil spill collectors

## Abstract

In this study, an easy, rapid and eco-friendly method was used successfully to synthesize the magnetite nanoparticles (MNPs). In order to fine-tune the synthesized MNPs for the collection of heavy crude oil spills, the particles’ surface was modified with green hydrophobic biocomponents that were extracted from *Anthemis* *pseudocotula* (AP). The surface modified reaction carried with that of the MNPs in the presence of *n*-hexane extract (APH) resulted in the formation of APH-MNPs, while in the presence of chloroform extract (APC), resulted in APC-MNPs formation. The as-formed MNPs were thoroughly characterized using transmittance electron microscopy, X-ray powder diffraction, vibrating sample magnetometer and thermogravimetric analysis. The efficiency of the surface-modified MNPs for the collection of oil spills in the presence of an external magnetic field was evaluated by taking different ratios of MNPs:crude oil. From the analysis of the results, we found that the APH-MNPs particles have higher efficiency in the collection of heavy crude oil than the corresponding APC-MNPs.

## 1. Introduction

Oil spills are one of the most severe marine environmental disasters, causing water pollution through the release of several hazardous chemicals into the environment. The removal of oil spills is expensive and costs billions of dollars [1,2,3]. There are many techniques that can be used to combat oil spills, including mechanical, bioremediation and chemical techniques [4]. Among these techniques, chemical means of separation have become one of the most common methods, where different polymers and copolymers are used to absorb, disperse or collect the spilled oil [5,6,7]. In addition to polymers, some ionic liquids, poly (ionic liquids) and surfactants have also been tested in recent years [8,9,10,11]. The removal of spilled oils by employing chemical collectors has become one of the most acceptable techniques because of its high efficiency and ability to reuse the chemicals as compared with other methods such as oil spill sorbents [9,12,13].

Based on new and restricted environmental regulations, the use of chemicals to combat oil spills might represent another source of marine pollution. These regulations prompted researchers across the globe to search for alternatives to traditional chemicals [14,15]. In that view, natural products can be rich alternative sources for several chemicals that have been used in different fields, including medicine, industry and nanotechnology. Recently, the biosynthesis of nanomaterials using ecofriendly, nontoxic and low-cost natural materials, such as plant extracts, fungi and biomolecules, have been extensively investigated [16,17,18,19,20,21,22,23,24,25,26,27,28,29,30,31]. Since natural materials act as reducing, stabilizing or capping agents during the synthesis of nanoparticles, employing such materials in the final product formation or surface modification steps can provide remarkable results. Many studies have reported the synthesis of magnetic nanoparticles (MNPs) using plant extracts; for instance, *Kappaphycus alvarezii* whole plant extract, plantain peel extract, a proanthocyanidin seed extract from grapes, *Carica papaya* and *Perilla frutescens* leaf extracts [32,33,34,35,36]. In our previous study, asphaltene that was precipitated from crude oil was modified and applied as a natural capping agent for the protection of MNPs from self-aggregation [12]. *Anthemis pseudocotula* is a member of the genus *Anthemis* from the Asteraceae family that grows widely in different regions of Saudi Arabia. This plant is a semi-prostrate, dense annual herb with bright, dark-green, feathery leaves and white flowers [37]. To the best of our knowledge, this plant has never been used for the synthesis of nanoparticles. Thus, the hydrophobic components from the aerial parts of this plant extracted by *n*-hexane and chloroform were applied for the biosynthesis of MNPs as capping and stabilizing agents. Furthermore, we evaluated the efficiency of the as-synthesized MNPs in the collection of oil spills using different MNPs:crude oil ratios.

## 2. Materials and Methods

### 2.1. Materials

All reagents that were used to synthesize the MNPs were based on ferrous chloride tetrahydrate (FeCl_2_·4H_2_O ≥ 99%), ferric chloride hexahydrate (FeCl_3_·6H_2_O, 97%), ammonium hydroxide (25%), ethanol, chloroform and *n*-hexane, which were supplied by Aldrich Chemical Co. (Missouri, USA) and were used without any further purification. Saudi heavy crude oil that was produced from the Ras Gara oilfield, Ras Tannora, and seawater that was collected from the western Arabian Gulf at the Saudi coast were used to simulate an oil spill.

### 2.2. Preparation of Plant Extracts

The aerial parts of *A. pseudocotula* were collected from a wild area of Rowdah Khuraim during March 2015 and were then identified by a taxonomist in the herbarium division of King Saud University. The collected fresh parts were chopped into small pieces and were air-dried in the shade. The dried material was extracted sequentially with *n*-hexane and then chloroform three times for 72 h each at 25 °C. The organic extracts were filtered and concentrated under reduced pressure and temperature conditions. The *n*-hexane and chloroform extracts were abbreviated as APH (*A. pseudocotula* hexane extract) and APC (*A. pseudocotula* chloroform extract), respectively.

### 2.3. Synthesis of MNPs

For the synthesis of APH-MNPs and APC-MNPs, Fe^3+^ and Fe^2+^ (2:1 Molar ratio, 5.406:1.988 g of each was dissolved in 100 mL of deionized water) solutions were added and stirred with the APH or APC solution (2 g dissolved in 100 mL of ethanol). Then, ammonia solution was added dropwise with continuous stirring at 25 °C. The solution pH was adjusted to 10, and the solution was stirred for 1 h to ensure homogenization and completion of the reaction. The produced APH-MNPs and APC-MNPs were separated easily using an external magnetic field. The MNPs were then washed several times with ethanol, followed by deionized water and, finally, were dried at room temperature.

### 2.4. Characterization

Fourier transform infrared spectroscopic analysis (FTIR; model Nexus 6700 FTIR, Thermo scientific, MN, USA) were used to investigate the functional groups of extracts and the synthesized MNPs. X-ray powder diffraction (XRD; BDX-3300 diffractometer, Beijing University Equipment Manufacturer, China) using Cu Kα radiation of wavelength λ = 1.542 °A was used to analyze the crystal lattice structure of the MNPs. Dynamic light scattering (DLS; Zetasizer 3000HS; Malvern Instruments, Malvern, UK) with a 633 nm He-Ne laser was applied to determine the particle size, dispersity index and zeta potential of the synthesized MNPs, using ethanol as a solvent. Thermal stability (TGA) of the synthesized MNPs was tested using a Shimadzu, DSC-60 instrument (USA) while heating the MNPs in the range 25–800 °C under N_2_ atmosphere at a heating rate of 10 °C/min. A drop shape analyzer (model DSA-100, Krüss GmbH, Hamburg, Germany) was used to determine the contact angle. For the measurements, a small amount of MNPs was dispersed in ethanol, was spread on the surface of a glass slide, and was evaporated in an oven at 50 °C to form a thin layer of MNPs on the surface of the slide. The contact angle between seawater and this layer was measured at room temperature. A transmission electron microscope (TEM; JEOL JEM-2100F JEOL, Tokyo, Japan) was used to confirm the surface morphology of the synthesized MNPs. A vibrating sample magnetometer (VSM; LDJ9600 in a field of 20,000 Oe, LDJ Electronics, MI, USA) was used to measure the magnetic parameters of the synthesized MNPs.

### 2.5. Application of APH-MNPs and APC-MNPs as Oil Spill Collectors

In a 500-mL beaker, 1 mL of Saudi heavy crude oil was poured over 250 mL of seawater. Different ratios of MNPs to crude oil, ranging from 1:1 to 1:50, were added and mixed slowly with the crude oil over the seawater for 1 min by using a glass rod. After 5 min, a permanent Nd-Fe-B magnet (4300 Gauss) was used to collect the dispersed crude oil spill. The remaining oil was extracted from the seawater by using chloroform. The efficiency of the MNPs in the collection of the oil spill was calculated using the following equation:

(1) $$\;{\mathrm{CE}\;(\%)\;=\;V}_{0}{/V}_{1}\;\times \;100\;$$

where *V_0_* and *V_1_* are the volume of the removed and original oil, respectively. The used MNPs were recycled after collecting them with an external magnetic field and washing them several times with chloroform.

## 3. Results

The present work aimed to use the defatted hydrophobic extract APC and APH of *A. pseudocotula* to apply as capping agents and to increase the hydrophobicity of MNPs with excellent dispersion in crude oil spills more than dispersion in sea water. Moreover, using plant extracts in the synthesis of MNPs increases their economic feasibility as environmentally friendly materials in the aquatic environment. Therefore, the application of the plant extracts to synthesize the nanomaterials was considered as a promising green method due to their biocompatibility, low toxicity and eco-friendly nature because the plants are found in abundance in nature. It was also attempted to characterize the chemical, crystalline structures, morphology, thermal stability, morphology and magnetic properties of the modified MNPs to investigate the effect of the plant extract on the dispersion and stability of MNPs in both aqueous and non-aqueous environments.

### 3.1. Chemical Structure of MNPs

**Figure 1:**
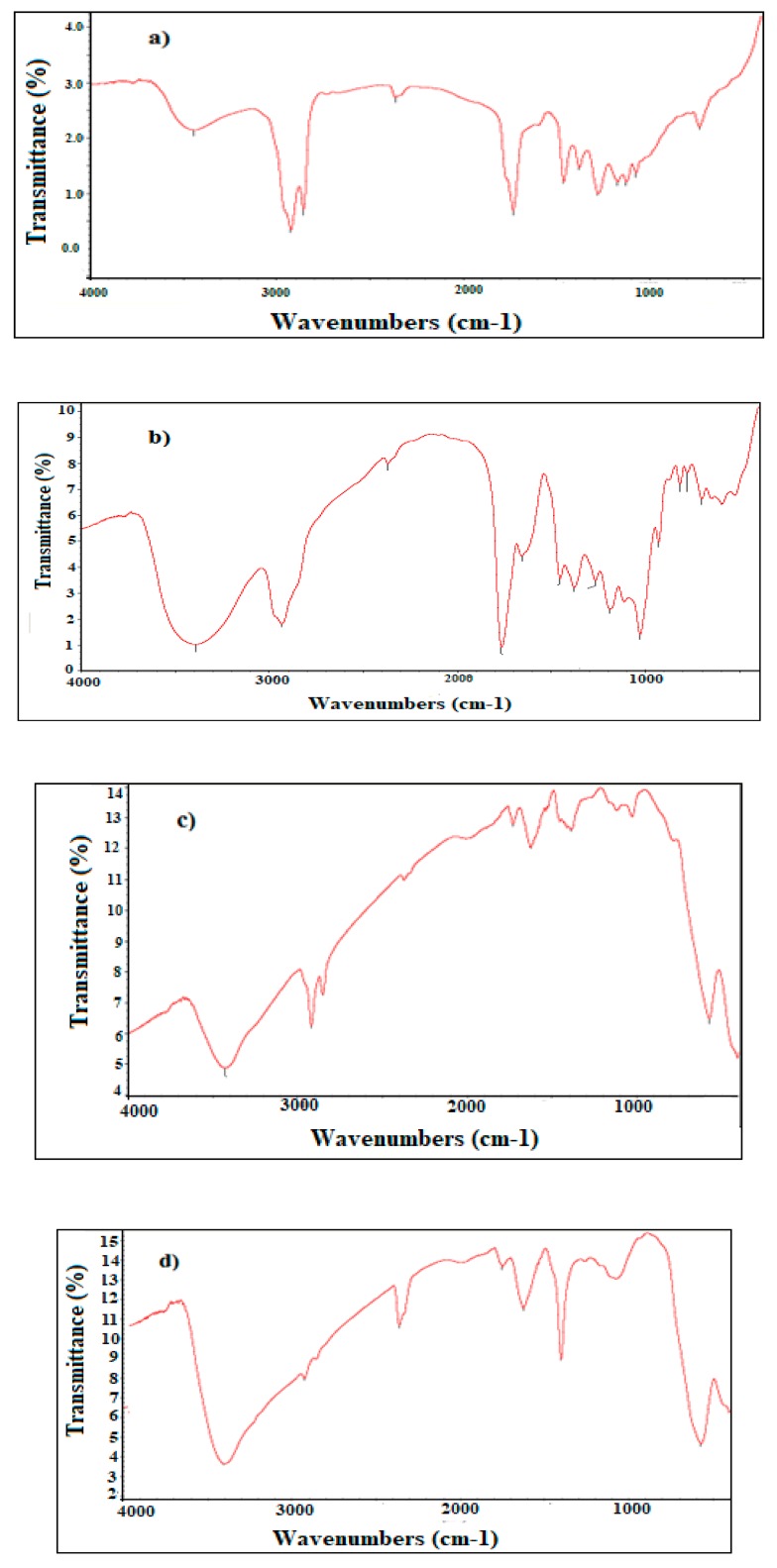
FT-IR (Fourier transform infrared) spectra of (**a**) APH (*n*-hexane extract), (**b**) APC (chloroform extract), (**c**) APH-MNPS (magnetite nanoparticles) and (**d**) APC-MNPs.

The active functional groups in both (APC and APH) extracts and the type of the produced iron oxide to synthesize MNPs (APH-MNPs and APC-MNPs) were investigated by FT-IR (Fourier-transform infrared) analysis, and the results are shown in Figure 1a–d.

**Figure 2:**
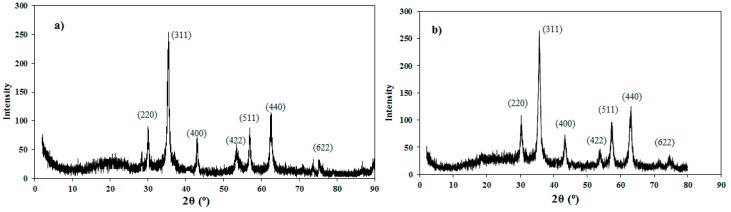
XRD (X-ray powder diffraction) diffraction pattern of (**a**) APH-MNPs and (**b**) APC-MNPs.

Figure 2a,b shows the crystalline structure of APH-MNPs and APH-MNCs, respectively using X-ray powder diffraction patterns.

**Figure 3:**
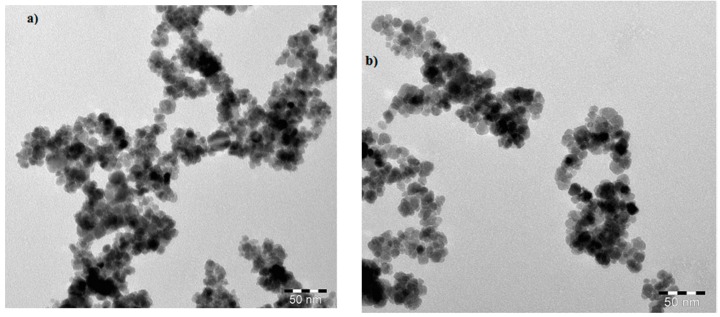
HR-TEM (High-resolution transmission electron microscopy) micrographs of (**a**) APH-MNPs and (**b**) APC-MNPs.

The surface morphologies of the synthesized APH-MNPs and APC-MNPs that were investigated by TEM are shown in Figure 3a,b.

**Figure 4:**
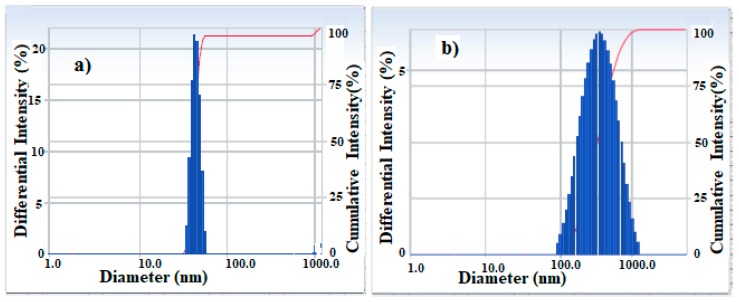
Particle sizes distribution of (**a**) APH-MNPs and (**b**) APC-MNPs in ethanol.

**Figure 5:**
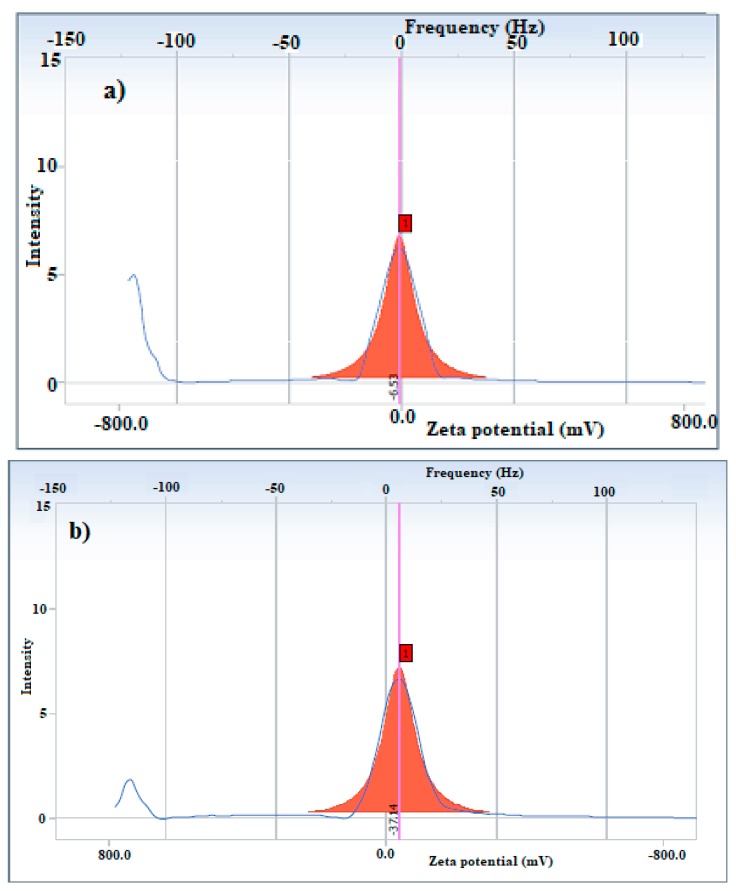
Zeta potential of (**a**) APH-MNPs and (**b**) APC-MNPs.

The DLS measurements that were applied for the determination of particle diameter, dispersity and zeta potential of the synthesized APH-MNPs and APC-MNPs in ethanol are shown in Figure 4a,b, Figure 5a,b and Table 1.

**Figure 6:**
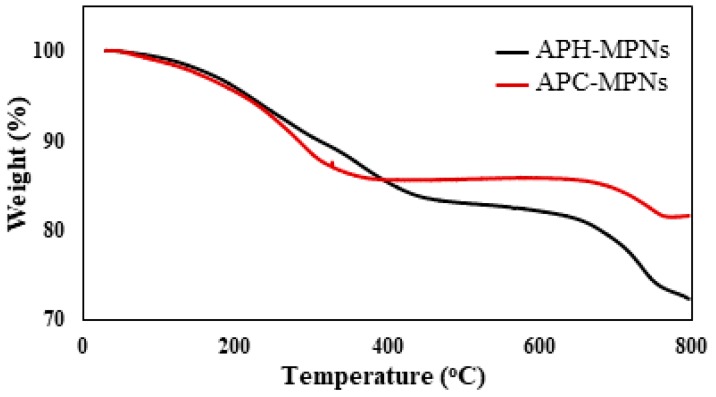
TGA (Thermal gravimetric analysis) thermogram of APH-MNPs and APC-MNPs.

The concentration and thermal stability of MNPs that were incorporated with the biomolecules extracts in the APC and APH were determined using TGA thermograms (Figure 6).

**Figure 7:**
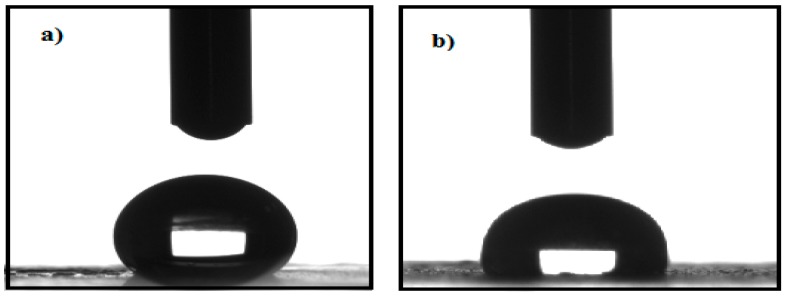
Contact angles of (**a**) APH-MNPs and (**b**) APC-MNPs.

In a general sense, the increasing of MNP’s dispersity in crude oil directly increases their efficiency towards the oil spill collection. Such dispersion of MNPs in crude oil is completely influenced by the hydrophobicity of capping agents, and in order to evaluate the hydrophobicity of the synthesized MNPs, the contact angle measurements were performed and are shown in Figure 7a,b.

**Figure 8:**
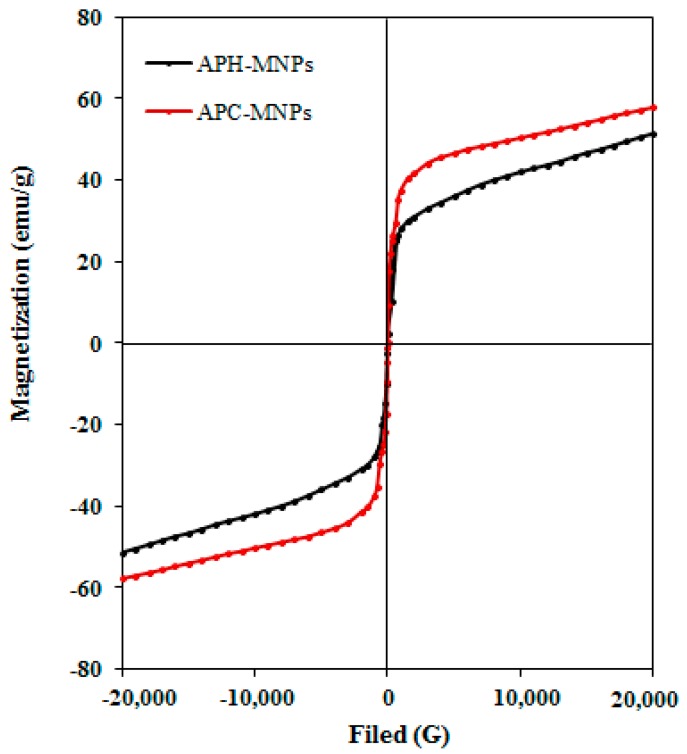
VSM (Vibrating sample magnetometer) hysteresis loop of APH-MNPs and APC-MNPs.

The magnetic properties of the synthesized MNPs, represented by the saturation magnetization (*M_s,_*), magnetic remanence (*M_r_*) and coercivity (*H_c_*), were determined at room temperature by VSM magnetic hysteresis loops. The results are shown in Figure 8 and in Table 2.

### 3.2. Efficiency of APH-MNPs and APC-MNPs in the Collection of Oil Spills

The efficiencies of APH-MNPs and APC-MNPs in the collection of an oil spill of Arabian heavy crude oil was evaluated at different MNPs to crude oil ratios (1:1 to 1:50) and are listed in Table 3.

**Figure 9:**
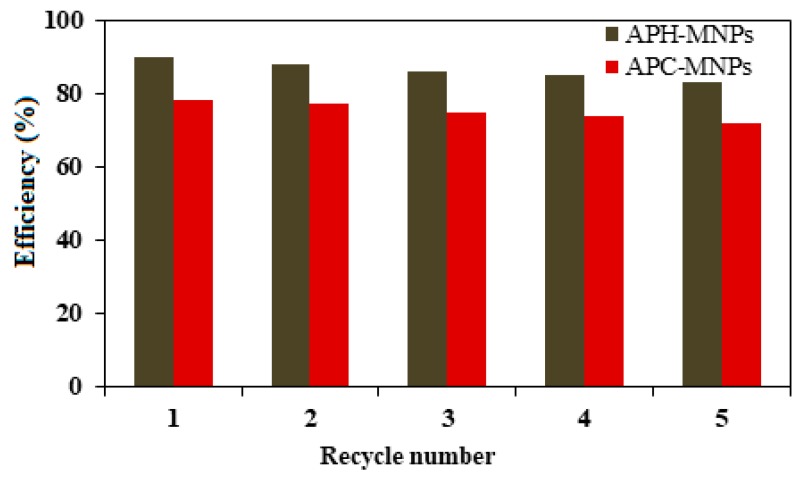
Efficiency of recycled MNPs in the collection of oil spill.

The reusability of the synthesized MNPs in the collection of oil spills was tested five times. After the collection of oil using an external magnetic field, the MNPs were washed with chloroform, followed by washing with ethanol and being air-dried, and were then used directly for the next run without further purification. The results for the recovered MNPs (1:10 MNPs to oil ratio) are presented in Figure 9.

## 4. Discussion

This work is aimed to investigate the efficiency of the green synthesized MNPs for the collection of crude oil in spills. The synthesis of MNPs was achieved using *n*-hexane and chloroform extracts of the aerial parts of *A. pseudocotula* as capping and stabilizing agents to increase the hydrophobic properties of MNPs. The wide availability of this plant, fast extraction process, low cost, green character and diversity of compounds in these extracts ensure their compatibility as capping agents for the MNPs. The *n*-hexane and chloroform extracts usually contain different active compounds, such as fatty acids, sesquiterpenoids, diterpenoids, phenolic compounds, coumarins and terpenoids [38,39,40]. The presence of these compounds in the capping agent increased their ability to form colloidal particles in the crude oil by utilizing several interactions, such as the aromatic π–π* stacking force, hydrogen bonding, Van der Waals force and electrostatic attractions. Accordingly, the use of these hydrophobic components as capping agents led to an increase in their dispersion in crude oil rather than seawater and further promoted their ability to collect oil from the surface of the seawater. The co-precipitation method is one of the most common methods that is used in the synthesis of MNPs [12]. Then, the MNPs can be formed via an oxidation and reduction method by using certain reducing agents, such as potassium iodide and sodium sulfite, followed by the addition of ammonium or sodium hydroxide after the removal of the precipitated iodine, as reported in our previous work [12]. In the present study, a mixture of ferric and ferrous ions (2:1 Molar ratio) were hydrolyzed easily in the presence of APH or APC, using ammonium hydroxide to form hydrophobic MNPs at room temperature. The overall reaction may be carried out according to the following equations:

(2) $$A.\;\mathrm{pseudocutula}+{\mathrm{Fe}}_{\left(\mathrm{eq}\right)}^{3+}+{\mathrm{Fe}}_{\left(\mathrm{eq}\right)}^{2+}+H_{2}O\;\overset{\mathrm{Stirring}}{\to }\;(A{.\;\mathrm{pseudocutula}/\mathrm{Fe}}^{3+}-{\mathrm{Fe}}^{2+})$$

(3) $$\left(A{.\;\mathrm{pseudocutula}/\mathrm{Fe}}^{3+}{\;-\;\mathrm{Fe}}^{2+}\right)\;+\;{8\mathrm{OH}}^{-}{}_{\left(\mathrm{aq}\right)}\;\overset{\mathrm{Stirring}}{\to }\;(A{.\;\mathrm{pseudocutula}/\mathrm{Fe}}_{3}O_{4}{)}_{s}↓↓{\;+\;4H}_{2}O_{\left(\mathrm{aq}\right)}\;$$

Magnetite can interact by complexation with heteroatoms such as oxygen and nitrogen that are present in the APC and APH extracts. The capping process might have taken place through a physical interaction, such as hydrogen bonding, Van der Waals, electrostatic attractions and/or polarity induction forces between the formed nanoparticles and various functional groups of phytomolecules that are present in APH and APC extracts [41]. The functional groups of the extracts (APH and APC) and the capped MNPs (APH-MNPs and APC-MNPs) were determined by FTIR spectra (Figure 1a–d), and the C–H stretching and bending vibrations of the aliphatic groups (CH_3_– and –CH_2_–) in the APH (Figure 1a) were observed at 2933 cm^−1^, 2856 cm^−1^, 1460 cm^−1^ and 1453 cm^−1^. Similarly, the appearance of bands at 3413 cm^−1^ and 1733 cm^−1^ indicated the presence of stretching vibrations of the polar functional groups –NH, –OH and –C=O, respectively. The same bands were appeared in the APH-MNPs spectrum (Figure 1c) indicating functionalization of these functional groups with MNPs. The appearance of a new band (Figure 1c) at 572 cm^−1^ (Fe–O stretching) confirms the formation of MNPs (magnetite) only without any other iron oxides. In the same way, the APC spectrum (Figure 1b) showed bands at 3413 cm^−1^, 2926 cm^−1^, 1748 cm^−1^ and 1402 cm^−1^ that referred to stretching and bending vibrations of –OH, –CH_2_ and –C=O. In addition, the appearance of other bands in the APC at 3139 cm^−1^, 1654 cm^-1^ and 2361 cm^−1^ was attributed to aliphatic =C–H, C=C and amide bonding, respectively. These active functional groups in the APC spectrum appeared also in the APC-MNPs spectrum (Figure 1d) with an appearance characteristic band of MNPs at 572 cm^−1^. An increase in the intensity of this band with capped MNPs in both samples (APH-MNPs and APC-MNPs) indicates an increase in the concentration of the MNPs. Moreover, a shift in the bands positions and decrease in the intensity of the functional groups in APH-MNPs and APC-MNPs (Figure 1b,c) also provides evidence for the successful bonding of MNPs with APH and APC functional groups. XRD diffractograms (Figure 2a,b) are used to investigate the iron oxide types beside the crystalline structure of MNPs [12]. The data (Figure 2a,b) showed several characteristic peaks at 2θ values of 30.13° (220), 35.48° (311), 43.15° (400), 53.95° (422), 57.03° (511), 62.62° (440) and 74.52° (622). These peaks were compared with the standard peaks in the Joint Committee on Powder Diffraction Standards (JCPDS) file (PDF No. 65-3107), and it was confirmed that the crystal structure of the MNPs was not affected by the modification of their surfaces with APC and APH components. The XRD patterns of MNPs (Figure 2a,b) elucidate the absence of diffraction peaks, i.e., amorphous nature, except for the observed broad band at 2θ values (20°–30°). The broad diffraction peaks (Figure 2a,b) appearing at 22.1° can be attributed to the presence of APH and APC phytomolecules, providing indirect evidence for the successful coating of the MNPs with APH and APC phytomolecules. Generally, the amorphous nature of the particles is non-toxic to living organisms and hence, amorphous herbal nanoparticles enhance the biocompatibility for different environmental applications [42]. XRD diffractograms were also used to estimate the average particle size of APH-MNPs and APC-MNPs by the Deybye-Scherrer equation. This equation depends on the relationship between particle size and broadening of the XRD peak and can be represented by the following equation:

(4)  PS = Kλ (βCOSθ) 

where PS is the particle size, K is a dimensionless shape factor called Scherrer constant (0.9), λ is the wavelength of X-ray (0.15406 nm), β is the width of the XRD peak at half the maximum intensity and θ is the Bragg diffraction angle. Using this equation, the average particle size of APH-MNPs and APC-MNPs were found to be 11–19 nm. The TEM micrographs of APH-MNPs and APC-MNPs (Figure 3a,b) elucidate the appearance of the MNPs in the cluster form which attributed to the magnetic behavior where the surface charges and magnetic forced them to be aggregated [9]. From DLS data (Figure 4a,b), the average diameter and polydispersity index (PDI) were found to be 565.1 nm and 0.338, respectively, for the APH-MNPs and 308.8 nm and 0.229, respectively, for the APC-MNPs in ethanol. However, considerable differences in the particle diameter as measured by TEM and DLS reflected the inclusion of agglomerated regions in DLS measurement that depends on the behavior of particles in the solution. Moreover, the average particle size that was obtained from the Scherrer equation supported the TEM results. Notably, the APH-MNPs were more agglomerated than the corresponding APC-MNPs, which might be caused by the Van der Waals attraction forces between the hydrophobic surfaces between these particles [43]. The small values of PDI confirms the formation of monodispersed MNPs that reflects the efficiency of APH and APC components as capping and stabilizing agents. The zeta potential of the APC-MNPs (Figure 6a,b) seemed to have a more negative value (−37.14 mV) than that of the AHP-MNPs (−6.53 mV), which further indicated the higher dispersity and stability of the APC-MNPs in ethanol as compared with the APH-MNPs. The concentration of the formed nanoparticles is determined from TGA thermograms (Figure 6). The magnetite contents of APH-MNPs and APC-MNPs were 72.5% and 81.5%, respectively, as determined at 800 °C. This indicated that there was a higher amount of capping agent on the APH-MNPs composite than the corresponding APC-MNPs composite. The data also elucidated that the degradation process seemed to have occurred at two different temperature regions, i.e., 100–400 °C and 670–780 °C. In the first region (100–400 °C), the APH-MNPs and APC-MNPs lost around 14% and 15% of their initial weights, respectively, and the losses could be ascribed to the decomposition of APH and APC groups. However, the weight losses in the second region were 10% and 3.5% for the APH-MNPs and APC-MNPs, respectively. The high thermal stability of APC-MNPs at the degradation temperature ranged from 400 °C to 700 °C, which might reflect stronger interactions between the relatively polar components of APC as compared with those of APH.

It was observed during the experimental studies that the synthesized MNPs exhibited no easy dispersion in seawater, while high dispersion was observed in toluene, xylene, chloroform and other low polarity solvents. The hydrophobicity of the prepared MNPs can be investigated from the contact angles measurements as represented in Figure 7. The data elucidated that the contact angles of APH-MNP and APC-MNP composites are 142° and 118°, respectively, which reflected the higher hydrophobic content of APH extract as compared against the APC extract. This means the formation of superhydrophobic capping of MNPs in the presence of APH extracts [42]. The magnetization curves for APH-MNPs and APC-MNPs composites (Figure 8) also indicate the superparamagnetic behavior. In addition, from the analysis, we observed an increased value for *M_s_* and decreased values for *M_r_* and *H_c_* as compared to other MNPs that were capped by different biocomponents [44,45]. The increase in the *M_s_* value of APC-MNPs reflected the lower amount of capping agent as compared with APH-MNPs, which was confirmed by the TGA analysis (Figure 6).

The contact angles measurements and magnetic properties of the synthesized MNPs show that increasing their hydrophobicity and supermagnetic nature make them suitable candidates in the collection of oil spills. Therefore, the efficiencies of different ratios of APH-MNPs and APC-MNPs as oil spill collectors for different MNPs: heavy crude oil ratios, from 1:1 up to 1:50 were evaluated. It is observed from the analysis that the best ratio of APH-MNPs:crude oil that succeeded in removing 90% of the crude oil was 1:10. In addition, the APH-MNPs were demonstrated to be highly efficient for the removal of crude oil in comparison to the corresponding APC-MNPs composite (only 78%). The observed high efficiency of APH-MNPs as compared to APC-MNPs reflected the greater hydrophobicity of the capping agent, which helped to increase its dispersion in crude oil, as confirmed by the contact angle measurements (Figure 7). Consequently, the efficiency of MNPs towards the collection of oil spills can be significantly improved by increasing the hydrophobicity of the capping agents. Moreover, the oil spill collection efficiency did not improve significantly by simply increasing the ratio of MNPs:crude oil to 1:1 because the MNPs could aggregate easily and, thus, formed aggregated clusters that disturbed the magnetic attractions between the MNPs and the external magnetic field. As observed in Figure 9, the efficiency of the recovered particles in the collection of the oil spill seemed to have decreased slightly with an increased number of cycles, indicating that the polarity changed as the material was reused.

## 5. Conclusions

In summary, hydrophobic MNPs could be synthesized in a novel, inexpensive, non-toxic and eco-friendly approach where the hydrophobic biocomponents extracted from *A. pseudocotula* were employed as capping agents. The surface functionality of these biocomponents with MNPs (APH-MNPs and APC-MNPs) and the persistence of the nanoparticles at all stages were confirmed by FT-IR analysis. The contact angle measurements provided proof for the effective dispersion of the synthesized MNPs in crude oil rather than in the water medium. The microstructures of the particles were supported by the TEM and DLS analyses where the particles were observed to be spherical in shape with an average diameter of 565 nm (APH-MNPs) and 308 nm (APC-MNPs). The superparamagnetic behavior was observed by the magnetic studies where the highest magnetization value was observed for the APC-MNPs as against the APH-MNPs. Such a decrease could be linked to the hydrophobic behavior. Furthermore, the APH-MNPs were demonstrated to be efficient for the collection of crude oil as compared against the APC-MNPs due to its higher dispersion ability in crude oil. Finally, the MNPs could be reused at least five times with no or slight loss of efficiency. Hence, by considering the simplicity of our synthesis method in addition to the wide availability of plant extract as a source for hydrophobic capping agents, the formed MNP-based composites can serve as potential platforms for the separation of oils from spills during seashore drilling operations.

## 6. Patents

Mahmood M.S. Abdullah, Ayman M. Atta, Hamad A. Al-Lohedan, Hamad Z. Alkhathlan, Merajuddin Khan, Abdulrahman O. Ezzat, Biosynthesized magnetic metal nanoparticles for oil spill remediation, Patent number: 9901903 USA(2018).

## Figures and Tables

**Table 1:** Dynamic light scattering results of APH-MNPs and APC-MNPs at 25 °C.

Sample	Particle Size (nm)	Polydispersity Index	Zeta Potential (mV)
APH-MNPs	565.1	0.338	−6.53
APC-MNPs	308.8	0.229	−37.14

**Table 2:** Magnetic parameters of APH-MNPs and APC-MNPs at 25 °C.

Sample	*Ms* (emu/g)	*Mr* (emu/g)	*Hc* (Oe)
APH-MNPs	51.42	0.153	6.4
APC-MNPs	57.83	0.098	5.1

**Table 3:** Oil spill collection results.

	Ratio	1:1	1:10	1:25	1:50
Sample					
APH-MNPs		92	90	88	83
APC-MNPs		81	78	74	70
